# Protective and Risk Factors of Italian Healthcare Professionals during the COVID-19 Pandemic Outbreak: A Qualitative Study

**DOI:** 10.3390/ijerph18020453

**Published:** 2021-01-08

**Authors:** Amalia De Leo, Eloisa Cianci, Paolo Mastore, Caterina Gozzoli

**Affiliations:** 1 Department of Psychology, Università degli studi della Campania Luigi Vanvitelli, 81100 Caserta, Italy; 2 Department of Psychology, Cattolica University, 20123 Milan, Italy; Eloisa.cianci@unicatt.it (E.C.); paolomastore@gmail.com (P.M.); caterina.gozzoli@unicatt.it (C.G.)

**Keywords:** risk factors, protective factors, COVID-19 pandemic, healthcare professionals, psychological malaise

## Abstract

The COVID-19 pandemic put the Italian health system under great stress. The sudden reorganization of work practices and the emotional impact of the large number of the victims had many consequences on the well-being of the healthcare professionals (HCPs) involved in managing the crisis. In the available literature, most studies have focused on the risk aspects while only few studies also take into account protective factors. For this reason, it was decided to conduct, within psycho-sociological perspective, a qualitative study with the aim to explore in depth the protective and risk factors as experienced by HCPs who worked in the Italian healthcare system during the COVID-19 outbreak. A total of 19 semi-structured interviews were conducted with HCPs–9 nurses and 10 physicians (7M and 12F) with an average age of 43 (SD = 13.4)–selected using snowball sampling. Considering three different levels of analysis the results highlight the protective and risk factors: personal history level (intrinsic/ethical motivation and flexible role versus extrinsic motivation and static role), interpersonal level (perception of supportive relationships with colleagues, patients, and family versus bad relationships), and organizational level (good leadership and sustainable work purpose versus absence of support from management and undefined or confused tasks).

## 1. Introduction

Following the Hubei region of China, that faced the coronavirus disease (COVID-19) since December 2019 [1], Italy was the first country in Europe to deal on a massive scale with the virus that, especially in the first phase, spread faster than in other areas [2]. The Italian healthcare system was not prepared to deal with the pandemic and the lack of knowledge on the virus and the absence of effective management models led to a climate of uncertainty and anguish that put the whole health system under great pressure.

The most difficult moments occurred especially during the first weeks of pandemic when HCPs had to deal with insufficient personal protective equipment (i.e., gowns, masks, and face shields) [3] which exposed the personnel to higher risk of contracting coronavirus than the general public [4] making it particularly vulnerable [4,5,6,7]. These objective difficulties were coupled with psychological difficulties linked to the fear of contracting the virus, infecting one’s family members, and, above all, to one’s sense of helplessness in the face of patient losses [8].

Within this scenario it is particularly important to analyze the psychosocial and organizational impact that the emergency is having on the healthcare system and the potential consequences on the mental well-being of the HCPs.

Most studies that have been published since the beginning of the pandemic agree that HCPs have been exposed to increased psychological distress [9] and to high risk of developing unfavorable mental health [10,11]. Prevalence of anxiety and depression symptoms was detected among operators [12,13,14] and significant levels of depersonalization and emotional exhaustion emerged between the most frequent burnout symptoms [15]. The malaise was often accompanied by physical symptoms, among the most common: fatigue, poor sleep quality, headaches, changes in eating habits and muscle tension [15,16,17]. PTSD occurred mainly as a result of a death of a colleague and the feeling of impotence in the face of the spread of the virus and its mortality rate [18].

Among the risk factors of developing unfavorable symptoms during the COVID-19 emergency, the literature highlighted some issues resulting from the organizational strategies. Especially in the early stage of the emergency the HCPs worked in the absence of clear intervention protocols, adequate instructions, and PPE [17]. In addition, several studies reported the relationship between HCPs’ psychosocial outcomes and organizational aspects, such as lack of clear communication from organizations [19], lack of support from colleagues [19] and inadequate organizational support (i.e., counselling and psychological support from the employer, and insurance and compensation) [20].

Moreover, some studies found that longer working hours [21], increased work intensity or patient load per hour [22], and heavy workload [23] can be considered additional risk factors.

As in any crisis scenario, the COVID-19 pandemic not only revealed the difficulties, but also the resilience factors raised from the individual and collective efforts against the epidemic. Previous studies highlighted that the most common coping strategies used by HCPs during an emergency are the acceptance of the critical situation and use of a positive prospective while working [24]. Perceived social/interpersonal support, higher self-efficacy, locus of control, and sense of coherence are among the most important individual factors in reducing stress and anxiety, preventing interpersonal deficits, and have been considered as significative protective factors [25]. Other scholars, focusing on the motivational factors that supported HCPs, found that financial remuneration was not considered significant to the medical staff.

Moreover, Khalid et al. [20] pointed out that a positive attitude in the workplace had the biggest impact in reducing staff stress. A clear communication and distribution of tasks, flexible working hours, the provision of psychosocial and psychological help without stigmatization and generally more training and organizational support for individuals are considered particularly important measures [9].

In other words, from an organizational point of view, a positive working environment and the assurance of individual safety while at work were the two main factors which enabled health professionals to continue working during the epidemic.

Analyzing the literature, we can see that most studies are quantitative research that focus mostly on the risk factors which affected HCPs caring for COVID-19 patients. Conversely, there are fewer qualitative studies that extensively explore the protection factors, in addition to risk. Furthermore, there is a lack of studies in the literature that have analyzed the Italian context-and in particular the Lombard one-although the region was the first and most affected by the health crisis caused by COVID-19 in Europe.

In the light of these considerations, our study aims to understand in depth the risk and protective factors in the perception of HCPs who worked in the Italian healthcare system during the COVID-19 outbreak. Considering our previous experience in the study of organizations and within the constructivism, we have chosen to adopt a psycho-sociological approach to the study of professional roles to achieve our aims [26,27,28].

The psycho-sociological approach considers professional identity as the interweaving of relationships between personal history, interpersonal relationships and organizational context at theoretical and operational level [29]. According to this theoretical prospective, three different levels of analysis have been considered:

(1) The personal level (the representation of personal history and professional role);
(2) The interpersonal level (the quality of the relationships);
(3) The organizational level (the representation of the management and the professional task).

The intertwining of these three levels allows us to carry out a more in-depth investigation and to grasp different aspects of the professional experience of HCPs. In terms of methodology, we opted for Interpretative Phenomenological Analysis (IPA) [30,31,32] because, due to the importance that it gives to the personal meanings attributed by participants to their experiences, it offers a sophisticated level of analysis.

## 2. Materials and Methods

### 2.1. Aims and Scope

This study, adopting a qualitative methodology, aims to explore the risks and protective factors in the perception of the HCPs who worked during the Covid-19 pandemic in the Northern Italy. In this study, we aim to better understand the factors that have a protective effect in situations of high emergency (in order to promote these aspects) and among the different risk aspects, identify those that are experienced as particularly stressful by professionals (in order to manage them).

### 2.2. Sample

The data collection process was conducted through semi-structured interviews with different health-care professionals (HCPs) who were working during the COVID-19 outbreak. The participants’ sample included 19 HCPs from emergency services, hospitals, nursing facilities, and private clinics.

By using a snowball sampling method, we involved 10 physicians (two general practitioners; three anesthetists; two geriatricians; one pediatrician; one surgeon) and nine nurses (all of them had worked in intensive care units during the outbreak) based in Lombardy. The snowball sampling enabled us to acquire new participants recruited by the already existing study participants. Specifically, we selected the institutions that were the most overwhelmed by COVID-19 patients in Milan, Brescia, Cremona, Mantova, Varese, and Como. The sample was made up of seven males (36%) and 12 females (64%), with an average age of 43, and a range of 40 between the oldest (65) and the youngest (25) interviewees (SD = 13.4). More specifically, among physicians 6 of them were males (60%) and 4 females (40%), with an average age of 47 (SD = 15.03). Furthermore, among nurses 1 of them was male (11.1%) and 8 were females (88.9%), with an average age of 37 (SD = 9.80).

The inclusion criteria were frontline HCPs who: (1) provided care to patients with COVID-19 symptoms; (2) worked in Lombardy during the pandemic; (3) voluntarily participated in the study. On the other hand, the major exclusion criterion was being unable to participate in the interview session during the study period. By interviewing HCPs who met the inclusion criteria, we reached the definitive number of respondents to obtain a sufficient level of collected data.

### 2.3. Measures

In line with principles of the constructivism, we developed a semi-structured interview which explored different thematic areas looking at risk and resource factors. The main interview questions posed to the participants concerned the representation of their personal history and professional role (personal level); the perception of the quality of their relationships (interpersonal level); and the representation of management and professional tasks (organizational level). We chose to adopt the Interpretative Phenomenological Analysis (IPA) [32,33] because the ideographic nature of the method allows to explore in depth the participants perspective and their personal view of world. The IPA method is characterized by an idiographic approach and focuses on specific details; this makes it particularly suitable for studying individual cases. Moreover, IPA considers participants the real experts of the topic being researched, so allows for phenomenological research in the attempt to give more importance to the subjective and interpersonal truth.

### 2.4. Method

When we conducted the interviews (July 2020) restrictive measures to contain the spread of the virus were still in place; thus, it has not been possible for us to meet the participants in person. For this reason, we decided to carry out the interviews through the web application Zoom. We shared with the interviewees the purpose and the meaning of the study in advance and scheduled the time slots for the interviews at their convenience. The interviews were planned as follows: all the physicians at first, all the nurses later. Before the individual meeting, a consent form and a socio-demographic anonymous schedule with a brief description of the research project was sent to each participant; they were asked to fill it out and return it by the day of the interview. The interviews were conducted by two researchers. All the sessions involved one interviewee at the time and took 50–60 min per person. If the participant exhibited emotional problems during the interview, adequate psychological intervention was provided to prevent secondary psychological harm. The interviews were recorded with the consent of the participants and fully transcribed. The research was approved from “Ethical Committee of Psychological Research, Department of Psychology, Catholic University of the Sacred Heart” on 20 July 2020.

### 2.5. Data Analysis

In line with our goals, we conducted a paper-and-pencil content analysis using the procedure described in the phenomenological interpretative approach, IPA [31]. The IPA aims to understand the experiences and explore the process of construction of meaning that individuals use in order to understand the subjective perspective and take into account the socio-cultural context in the data interpretation process [30,32,33]. Therefore, all the interviews were transcribed verbatim and analyzed individually and the different stages of the analyses were cross-checked at various intervals by two authors. The researchers reviewed the interview materials, summarized and extracted meaningful statements, and formulated the present themes. Conflicting opinions on the contents of a theme were discussed and resolved.

We proceeded with a hierarchical categorization process combining a top-down and a bottom-up logic. Specifically, after having defined the three macro-categories (“individual history,” “group and relationships,” and “organization”) consistent with the psycho-sociological approach on the study of professional roles, we extrapolated, through a bottom-up logic, different micro-categories that emerged directly from the words of the HCPs. Following these phases, we started an interpretative process in order to explain the relationship between macro and micro-categories.

## 3. Results

**Figure 1:**
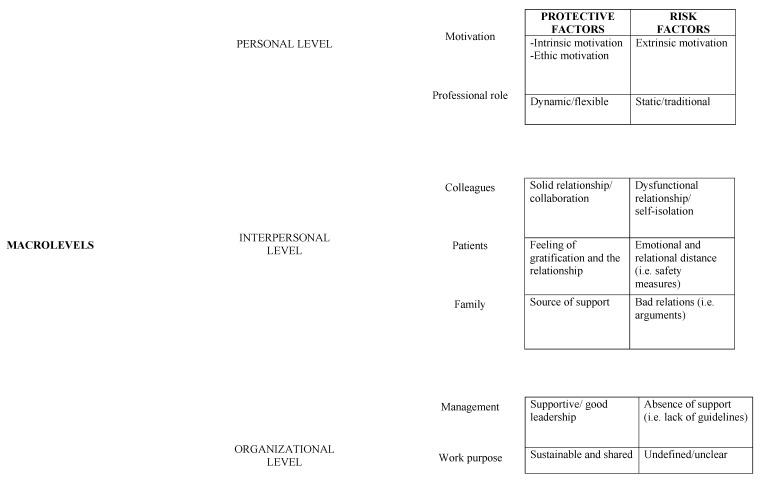
Data structure.

According to the objectives of the study, the cross-sectional and shared key issues between the two professional roles analyzed (physicians and nurses) that arose from the interviews will be presented herein. In particular, we will report the different subordinate themes that emerged from each of the 3 levels considered–personal history, interpersonal relationships and organizational context–looking at the risk versus protective factors (Figure 1). It is important to specify that in relation to our research goals, during data analysis we found no relevant differences between physicians and nurses (except for one category), so, after a first step of differentiated analysis, we decided to treat the two categories together.

### 3.1. Personal History

As for the individual level, two sub-categories emerged from the bottom-up analysis: motivation and the type of professional role.

#### 3.1.1. Motivation

Three different kinds of motivation arose from the narration of the participants: intrinsic, ethical, and extrinsic.

In thirteen cases, the narration of the professional experiences of the participants revealed that an intrinsic motivation was the foundation of their choice of profession.

The representation of one’s role with a social value, as a choice based on an inner desire, and an individual propensity to care for others constituted the most relevant protective factors.

*“Since I was young, I have always wanted to be an oncologist, because my grandfather died of cancer. He also bought me a doctor’s case… Epiphany!”*

*(Physician)*

*“… I chose to be an intensive care doctor because during my postgraduate studies I was working in an emergency ward and I was fascinated by the intensive care doctors I met. I was attracted towards them when, walking onto the first floor where the intensive care was, they didn’t speak, they always understood each other silently just with some gestures... they looked like angels. I was fascinated by this”*

*(Physician)*

*“I wanted my life to have a noble mission. What is nobler than helping people?”*

*(Physician)*

*“It was what I have always wanted to do, my dream was to work in surgery, and I reached my goal… surgery is my industry, I love my job and it is a great source of satisfaction”*

*(Nurse)*

*“When I saw the nurse, who was taking care of my sick sister at home, I realized I would have to become a nurse too”*

*(Nurse)*

The most critical factors which motivated the medical staff to continue working during the crisis, even when their own life was at stake, were their social and moral responsibilities and their professional obligations [34]. The interviews revealed that the respondents’ moral sensitivity was responsible for a more solid professional resilience:

*“There was a reason I was there. That’s what made me do what I was able to do and what I had studied for, although it was very difficult at that time”*

*(Physician)*

*“I accepted the risk of contracting the infection by continuing with my job. I could have found some justifications to stay at home”*

*(Physician)*

*“There was a clear risk, but we had to worked anyway and use the available resources to the best of our ability. You have to do what you need to do; you don’t have a choice you are aware that your dedication makes sense”*

*(Nurse)*

*“I felt I benefited from being there as a human being. I don’t work for money”*

*(Nurse)*

*“The patient comes first. That’s how I am!”*

*(Nurse)*

In our sample, however, there were also three interviewees who were not entirely sure about pursuing a career as a doctor or nurse; rather, they found themselves following this professional path a little by chance, or by fallback (extrinsic motivation).

In this case, the COVID-19 outbreak represented the trigger which brought to light some uncomfortable factors which might have already been present at a latent level. This element was one of the most important risk factors for the interviewed healthcare professionals during the outbreak:

*“… I wanted to be a teacher or a journalist… I completely changed my mind. Then I tried the MCAT” *

*(Physician)*

*“…For the healthcare operators who have chosen this job because they didn’t find anything else it was harder”*

*(Nurse)*

*“I don’t know if I will always feel this sense of isolation at work, I don’t feel I am part of the system. I think I will choose to become a pediatrician in another context”*

*(Physician)*

#### 3.1.2. Professional Role

In our sample, two different representations of the professional role emerged. In the middle of the crisis, those who considered their professional role as dynamic and flexible expressed that they were able to more easily adapt to the different needs and capabilities required by the pandemic, living the experience as a personal challenge. Conversely, the five participants who considered their job in a more static and traditional way made a greater effort in dealing with the emergency.

In addition, less working experience and the self-perception about a lack of competency to take care of COVID-19 patients were associated with an increased perception of stress and burnout [35]:

*“Nurses like me, working in surgery, we changed our job in one day. We suddenly moved from surgery to intensive care unit, and this constituted a significant distress. Indeed, our distress didn’t derive just from the patients with their new disease we faced, but also from the new tasks we had to undertake from one day to the next. A shared consideration was the fear of not measuring up to this situation and to the patients. A relevant anxiety factor were thoughts like this “will I be able to manage this situation and truly help my patients? Couldn’t I potentially worsen the patients’ conditions given my little experience in that specific field?”*

*(Nurse)*

*“We felt (helpless) like flies”*

*(Physician)*

*“The old-fashioned doctors who are used to having reception staff, were now without a secretary, unable to write an email or to send a text–I covered for a doctor who didn’t have a mobile phone–Probably these people had more difficulties than others, especially in changing their working approach”*

*(Physician)*

*“We concretely did a new job without the correct knowledge of it”*

*(Nurse)*

*“I changed my working approach: I’m a geriatrician, but during the outbreak I became like a doctor who works in hospital. These two professions are different, I’m not a hospital doctor, I’m a geriatrician, I have not got these clinical competences. I know geriatrics and professional ethics, for example. I had to forget these competences”*

*(Physician)*

In brief, as regards this level of analysis, the main protective factors that have emerged are the intrinsic motivation, the social responsibility of the HCPs, and a more solid and therefore flexible professional identity, adaptable to needs and emergencies that were not perceived as disqualifying.

The risk factors identified are an original de-motivation to join the profession and a more technical and rigid conception of the role. What puts the operators most in crisis are therefore the demands for changing their professional practices perceived as devaluating.

### 3.2. Interpersonal Relationships

As for relationship dynamics, three nuclei emerged: colleagues, patients and family.

#### 3.2.1. Colleagues

According to twelve cases of our sample, working in a state of emergency consolidated or facilitated the relations among colleagues.

The COVD-19 pandemic caused an important restructuring in terms of relationships in the working context giving more value to teamwork and cooperation among colleagues. The respondents reported what fostered this revival were the changes seen in colleagues and in particular a management that has been able to support and enhance professionals in carrying on the new tasks. They also underlined the importance for HCPs to work effectively in teams with a multidisciplinary approach. In particular, some of them argued that it has been possible to treat patients with COVID-19 symptoms in an efficient way only thanks to effective teamwork and the sharing of different points of view, in terms of competences and knowledge:

*“Probably all of us had the same difficulties, we were in the same boat, we helped each other, and we formed a group, that isn’t easy in general. The birth of a group was very positive, I don’t know why, maybe we were very lucky, but certainly this fact helped all of us”*

*(Nurse)*

*“We discussed every situation in the group, we didn’t work individually like Rambo… This fact was essential because it helps you to respect your position and because everyone could have his/her own idea about a situation”*

*(Physician)*

*“Collaboration was stronger and more supportive, we removed personal matters… In my experience, due to the necessity, teamwork improved”*

*(Physician)*

*“I think few people consider this experience in a positive way; I am one of them. This experience was very satisfying”*

*(Nurse)*

*“Yes, we collaborated a lot, especially nurses. Before the outbreak there were two groups, during the outbreak and also now we have formed a single group that work together to face this situation”*

*(Nurse)*

Conversely, three of the HCPs saw the relationship with colleagues as an element of further difficulty. Overall, those with a feeling of self-isolation and lack of support from colleagues were the most exposed to distress. A bad relationship with colleagues not only represents a non-protective factor, but it can also be considered one of the major risk factors for the dissatisfaction of HCPs who worked during the COVD-19 emergency. In these cases, the crisis caused by COVID-19 represented a “detonator” of some already existing difficulties and conflicts. It should be highlighted that in these cases the top management has not been able to support a good team climate and enhance workers during the crisis.

*“This is a crucial point, the integration of a new person in a group is usually very difficult. I haven’t joined the group yet, I understand the organizational policy, I understand my role in the team, but personally and relationally not yet. I’m still excluded, in particular I work with two people, and nowadays I have a solid friendship just with these two colleagues, therefore I don’t relate with 80–90% of the group”*

*(Nurse)*

*“In the end people always reveal themselves for who they are, because as soon as things tend to improve, as it seems they are doing now, they return to what they were. And, unfortunately, some people–maybe even me, since I can’t see myself from the outside–get even worse!”*

*(Physician)*

*“Every morning we were angry, everyday there was a girl, operator, or nurse, who screamed and cried, because it was very difficult working together in this situation…”*

*(Nurse)*

#### 3.2.2. Patients

Certainly, the most essential factors that motivated the medical staff to continue working were their social and moral responsibilities towards their patients.

The feeling of gratification and the relationship with the patients constituted the driving force to go on for twelve of our participants (motivational factor to face the crisis which in many cases pushed the HCPs also to put their own lives at risk). From our interviews, it emerged that all the safety measures, the social distancing, and the personal protective equipment (mask, face shield, and gown) caused an emotional-relational distancing which had a huge impact on the HCPs’ professionality. Nurses, who attributed a primary importance to the relationships with the patients, were especially impacted.

*“A. was the first patient who woke up, I think that I will never forget this episode. When I saw him awake, I asked him: “What kind of music do you like?” he told me–or rather he wrote on a sheet of paper because he was intubated–that he is a V.R. fan, therefore, that evening, we heard a V.R. concert… it was a fantastic moment. His recovery gave me hope and the strength to carry on. For this reason, I thank him”*

*(Nurse)*

*“It was a drama, because also the patient not at risk fell into depression. Despite me, I’m joyous and playful with old patients, I love my job, but with the mask and the gloves, patients didn’t recognize you”*

*(Nurse)*

*“In this situation some operators will be justified when they say: “don’t touch me and don’t speak to me.” Instead, the relationship with patients must be the basis of everything. It’s fundamental that patients trust you, and can communicate with you”*

*(Nurse)*

Our sample included some interviewees who put in place a series of self-defense strategies to avoid being overwhelmed by the deep emotions caused by that type of emergency (feelings of helplessness): this is especially the case of the nurses who put the relationship with the patient at the core of their professional role.

*“Truly, I tried not to remember names and to avoid entering into a relationship”*

*(Nurse)*

*“It was very difficult, the first month to feel better, I didn’t look at the faces of the patients. Watching their face would be devastating for me. This fact would have meant giving an identity to the patient and thinking about his or her story. This was a way to put a wall in front of my emotions”*

*(Nurse)*

*“There was a time where I didn’t like my work and I didn’t like the working method: before COVID-19 you supported the patient, the nurse brought comfort: “Stay quiet, today you are here, could be worse, there is hope, think positive.” During the outbreak I couldn’t say this to my patients, who am I? Am I a technician? From this point, I didn’t like my work”*

*(Nurse)*

*“Operators, who usually talk to patients, were not recognized by patients, due to the mask and the cap. For example, patients mistook a girl for a man. We were all equal in their mind”*

*(Nurse)*

*“The relationship with patients was complex, because primarily non-verbal communication was absent. After some days we started to write our name on our shirts. Patients didn’t have a way of knowing who they had spoken to five minutes before. They couldn’t recognize us; we were all the same”*

*(Nurse)*

#### 3.2.3. Family

For fifteen participants of our sample, family and, more generally, loved ones represented a fundamental source of support for the HCPs.

*“Clearly family helped me a lot, they supported and sustained me”*

*(Physician)*

*“Coming back home after a difficult shift … the comfort of your family helped you a lot, we didn’t close the barriers completely: we maintained a hug or caress, I think these helped us”*

*(Nurse)*

*“On the familiar point yes, the support that I received was nice and enjoyable”*

*(Physician)*

Nonetheless, not all the participants lived the same experiences. That is the case, for instance, for a physician who during the emergency broke up a close relationship.

*“COVID-19 had a negative impact on my life, it caused the end of a relationship, due to the quarantine. It wasn’t too easy to seek support from people who weren’t living the same experience, it created a distance”*

*(Physician)*

*“Many times, my husband told me to leave this work because we have some savings. I don’t work for money, we fought a lot, because he wanted to convince me to stay home. I understood his position, we have a little child”*

*(Nurse)*

From most interviews, it emerged that the crisis consolidated their relationships and cooperation among colleagues representing an important relational source; unfortunately, in some cases the emergency management–especially at the most critical moments–generated episodes of disagreement and conflict among colleagues becoming a risk factor.

For most professionals, the relationship with patients represents an important source of motivation and gratification, but it can also mean a potential risk factor with regard to the management of physical and emotional fatigue. It is interesting to underline how for many HCPs the protective devices have had not only a physical protective function but also emotional, acting as “spacers” from the anguish generated by the suffering of patients.

As for the family relationships, most HCPs stated that family members understood the situation and supported them during the crisis; in some cases, however, misunderstandings and tensions–generated especially by the situation of uncertainty (e.g., fear of contamination)–occurred affecting negatively the well-being of HCPs. In other words, professionals were faced with the difficult decision to choose whether to protect their family or continue to work in a high-risk situation to saving lives.

Concluding, regarding this level of analysis, we can say that the crisis has had three types of implications over the interpersonal relationships: in the first case it consolidated the positive relationships already in place; in the second case it brought out conflicts and pre-existing misunderstandings; in the third, it gave the way to the emergence of new interpersonal relationships and, especially in cases where management was able to foster cooperation and mutual support, it led to new meanings and a relaunch of the relationships among colleagues.

### 3.3. Organizational Context

#### Perception of Organizational Support and Work Purpose

As for the organizational level, the most important factors that were reported as common triggers of stress were the lack of infection control guidelines and protocols, the lack of access to personal protective equipment, and the absence of support and appreciation by supervisors [36] and more in general by the management of the hospitals [37]. Furthermore, working in a different department, the changing of roles in order to cope with the emergency, and taking care of patients with a disease that had not been treated before, and in general the changing of the work purpose, led to a large number of negative emotions such as inadequacy, incompetence, and emotional destabilization.

*“In our field, we were forgotten. Nobody provided us with guidelines or protocols until April. We took the first swab in April, therefore there was a long delay from the outset of the virus”*

*(Physician)*

*“The feeling was that at the beginning, even at the highest hierarchical levels they found themselves displaced, we felt a little thrown into the storm without a life jacket. It took a while, maybe too long, to start seeing that something was moving, that the organization was bearing fruit, that we were not running out of drugs, that we were not running without devices, that everyone was doing their part”*

*(Nurse)*

*“I was very angry. Nobody (management) knew how to manage the situation. However, sometimes a comforting word could help. That was not the case, they answered one after the other without instructing you on what you could/should have done”*

*(Nurse)*

*“We did a different job, where we did not know a lot of things. For example, if I am in the emergency room and a patient who needs blood comes, I don’t know where to find the forms and I don’t know what to do, because this isn’t our field of work. Therefore, that really throws you, maybe this is the hardest thing now”*

*(Nurse)*

On the other hand, six of our professionals who recognized their leadership’s good management of the crisis despite the difficulties, experienced less feelings of isolation and felt more protected. In addition, the collective efforts against the epidemic, and the need to share knowledge in order to effectively treat COVID-19 patients led to a flattening of the hierarchies among doctors and nurses and to a greater collaboration among the different professional figures. This, together with a clear representation of work purpose, turned out to be an important support from the organizational point of view that has been useful in preventing much negative emotion such as fear, anxiety, and helplessness.

*“Our director was smart, because he directly got involved in the “game”; he ordered the professional masks too”*

*(Nurse)*

*“In the COVID department a hierarchy doesn’t exist, this concept was clear from the beginning. The operator follows the directive of the nurse; the nurse follows the directive of the doctor. We do our best, in line with our competences, but there isn’t a hierarchy, everyone collaborates. Everyone has a voice in the decision making and we discuss goals together, we collaborate a lot”*

*(Nurse)*

*“I read in some interviews about COVID, that this outbreak invalidated previous professional experience. I, with 2 years of experience, and a person who has been working for 15 years, and a new graduate are at the same level. In my previous experience I met doctors who said: “I make all the decisions,” instead in this emergency the nurse also has a voice in the decision making. This brought me pleasure, because for the first time a doctor recognized my professional position”*

*(Nurse)*

*“We weren’t unprepared professionally, because our competences were sufficient to face the situation”*

*(Nurse)*

*“There weren’t a lot of changes in the hospital environment. You take care of the patients, you help them, these are the goals of hospital recovery, this is the working method”*

*(Nurse)*

At the organizational level, the perception of HCPs was, on the one hand, to be protected and emotionally supported by a management able to contain the anguish and that tempts day by day to make the work purpose as clear and defined as possible; on the other hand many HCPs reported a perception of absent leadership associated with an unclear and confused work purpose. This was accompanied by feelings of confusion, loneliness, meaningless, and fear, and thus a greater risk of organizational malaise.

With the aim to synthetize the results we report below two tables that summarize the risks and resources factors described (Table 1 and Table 2).

## 4. Discussion

From a psycho-sociological perspective and thanks to a qualitative method, this research aims to explore protective and risks factors of the HCPs who had worked at the frontline during the outbreak of COVID-19 in Italy.

### 4.1. Risk Factors

In this study, we found several risk factors that could cause discomfort in HCP. At personal level, it emerged that a professional choice which is not directly related to the emergence of a desire or a propensity to care–extrinsic motivation–led to a higher risk to develop discomfort/malaise among HCPs. Probably they hadn’t been able to find the meaning of their own work during the crisis. These people might already have a hidden form of discomfort in the workplace which the COVID-19 outbreak definitely brought to light. As regards to the professional role, people that considered their role in a static way and that tend to rigidly adhere to the formal organizational mandate, tend to accept the changes caused by the crisis with more difficulty and fatigue. In this case it emerges a perception of oppression and a lack of organizational and social recognition. They describe, in fact, the situation with negative feelings, like inappropriateness, incompetence, and inadequacy. In short, role rigidity and the lack of concrete and shared objectives had led to less adaptation to change demands that come from the COVID-19 outbreak and thus had exposed HCPs to a greater risk of discomfort and professional malaise.

The interpersonal level (second level of analysis) includes the horizontal relationship that HCPs had with patients, colleagues, and family. Patients have a central role in the experience of HCPs, but the relationship with them represents an advantage only when the HCPs have the capacity and resources to approach the suffering of others without being totally absorbed or overwhelmed. In accordance with literature [38], from our sample emerges that especially in the first phase of the outbreak, HCPs used a lot of defense mechanisms that helped them to manage the huge emotional impact and the sufferings that this situation brought. Nevertheless, like any other psychological function, a normal defensive process can become pathological if abused or inadequate for age or situation. As for HCPs, inadequate defense mechanisms or an excessive use of these strategies might lead to less adaptation and to an impairment of mental health and job success.

As for the horizontal relationships, crisis, if on one hand reinforced the connection between colleagues, on the other hand, it contributed in worsening the already unstable and compromised relationships among colleagues. Especially in cases where organizations fail to ensure a favorable climate, feelings of loneliness, insecurity and sense of uncertainty prevail contributing to exacerbate situations of conflict and malaise among the staff. In this regards, different studies agree about the importance of a good team climate [39,40,41] in reducing the negative effects of the sense of isolation [42,43]. The personal link with family is a crucial point in the HCPs experience. The most of HCPs have shown feelings of fear about the potential transmission of the virus to members of their families and, in some cases, social distance, anxiety, and similar feelings threatened established deep relationships. This has contributed to the deterioration of the personal well-being.

In relation to the organizational dimension, a prevalent perception of an empty space, in institutional terms, emerged. Especially in the first phase, healthcare systems and primary care organizations were not prepared to face COVID-19 pandemic and were not able to guarantee HCPs’ primary safety (i.e., lack of protection devices or shortage of rules and protocols). The common dissatisfaction towards the working environment was also caused by the uncertain procedures to follow, established at the organization level [44]. This is the case for those professionals who had to re-signify their role (new tasks, change of unit) to the new needs required by the crisis.

### 4.2. Protective Factors

Given the scale of the pandemic, literature focused mainly on the negative aspects. This work opens the way to the study of the protective factors for the mental health of HCPs during and after the pandemic event. For this reason, we highlighted in addition to the risk factors, the resource factors that helped to protect HCPs.

An intrinsic or ethic motivation (described as social and moral responsibilities) and professional obligations, helped HCPs during the most critical moment of the outbreak (i.e., when they did not have the protection devices required and they did not have much knowledge about the virus) and encouraged them even if their own lives and families were at risk. In cases where the role was experienced as a personal choice, the fatigue that emerges in managing the crisis was balanced by focusing attention on pursuing the primary objective of “ensuring the well-being of the patients” [45]. A professional choice, based on the aid dimension, helps HCPs to restore sense and meaning to their work purpose, acting as a protective factor even in the most difficult moments. Correlated to the motivation, two different representations of the professional role were highlighted: dynamic/flexible and static/traditional. HCPs who consider their role as dynamic and flexible were more ready and prepared than other people to face the demands that the pandemic scenario required. For example, they can accept without any difficulties to change their professional role, their field of action or to collaborate in a new working team. These people describe this situation and these changes with positive emotions. One respondent reported, in fact, that the pandemic experience developed his professional competences and that he considered this period as a positive personal challenge, for instance. This general disposition of commitment, control, and challenge seems to be associated to the concept of “hardiness”. Hardiness is an individual attribute associated with resilience and it tends to be associated with positive internal states, leading people to consider external events as a challenge and an opportunity for self-improvement [46]. Individuals with a higher level of hardiness experience lower levels of stress and secondary trauma [47,48]. Nevertheless, this positive attitude depends not only on an individual disposition but also on a greater or lesser discretionary in interpreting one’s professional role, which is itself influenced by the organizational culture.

Patients, colleagues, and family represent an important source of relational and support for HCPs. The relationship with patients, in particular represents an important motivational source to persist in their work, also during the time of the crisis, especially for nurses. Data analysis revealed that for the most themes there are no significant discrepancies between nurses and physician, except when it comes to the relation with patients. This is probably because of the close and direct contact-requires by the professional role-that nurses have with patients. The relationship with patients for most professionals is a source of gratification, and those who demonstrated a significant investment in the relationship of care seemed to be more satisfied and encouraged to carry on their work. In addition, from our research, as already highlighted in literature, emerged that HPCs adopted avoidance and other similar psychological defenses in order to psychologically adjust to the situation. It is demonstrated that the adoption of these coping measures during stressful situations, like a pandemic, can alleviate stress and promote mental health (2020) [49].

The relation with colleagues is a necessary source of support [50] and represents an important resource factor. Emotional support provided by colleagues helped HCPs to prevent feelings of isolation; collaboration and sharing of knowledge guaranteed a greater effectiveness in their work and therefore a sense of greater self-control that was a protective factor against feeling of ineffectiveness and impotence. As for the relationship with family, although HCPs are afraid about the potential transmission of the virus to members of their families the relationship with family, and in general relationships beyond the working environment, constitutes fundamental protective factors. Parents, children, and friends are perceived as an important source of emotional support, like a shelter to find relief in the hardest moments. This means that in the perception of HCPs, relatives were able to understand their position and to support them even with small acts of care, such as an embrace or a word of comfort at the right time. This is in line with existing scientific literature, which suggests that the perception of having a significant life is associated with lower stress, more healthy behaviors and more active and adaptive coping [51]. In short, positive emotions are generally generated by a good relationship with colleagues, with family, and with patients.

Data analysis showed that although in most cases HCPs suffered from a lack of clear and specific intervention protocols and guidelines, in some cases, the vertical (organizational) dimension represented a source of support and it was perceived in a positive way. A good organizational climate and the perception of a sustainable and shared work purpose facilitate a collaborative behavior and integration among the different professionals. These findings enrich few studies that adopt an organizational perspective. They pointed out that it is important for health care organizations to appreciate the powerful adverse effects of internal factors such as leadership, management styles and administrative policies and to foster a healthy organizational culture through thoughtful attention to communication, relationships, self-awareness and the symbolic significance of policies and behaviors [19,52].

### 4.3. Strengths and Limitations

Certainly, the most important advantage of this study was to investigate at a deep level the experience of the HCPs who worked in contact with COVID-19 patients during the emergency in Italy. The study was carried out in the first phase of the COVID-19 outbreak and during this period participants were able to narrate what happened in a true and involved way. This has made possible to collect “fresh” and authentic data. In this regards it could be interesting to do a follow-up with the aim to investigate if there are any differences in the representations of HCPs once passed the first phase of the pandemic.

This study, of course, is not free of limitations. The approach used highlights the critical issues related to the analyzed context; therefore, the results obtained reflect what happens in a specific context and at a specific time. We suggest that future researches could involve other contexts and professionals in order to further explore the risk and protective factors that emerged in this study. According with our aim, we maintained the focus on the cross-sectional and shared elements between the two professional roles (physicians and nurses). In future studies, it could be interesting to make second level analyses in order to grasp the differentiating aspects between the roles also considering other variables (i.e., age). In our work, we selected participants giving priority to HCPs who worked closely with COVID-19 patients during the emergency; this led to have not a complete control over the age-factor and therefore to a large age range (25–65). In the future, differences due to age-factor may be further explored.

Finally, it would be interesting to deepen the fundamental role played by the management in order to identify specific actions useful to manage situations of extreme stress and emergency.

## 5. Conclusions

The rapidity of COVID-19 transmission, the concentration in health care settings and the impact on HCPs has highlighted the need for reviewing existing health care practices, procedures and organizational culture. Often, stress and malaise in the workplace has been seen as primarily the responsibility of the individual worker, and support strategies are focused mainly on improving the individual’s ability to cope with stress [19]. This study shows that the factors that contribute to creating situations of stress and organizational malaise are many and must be treated at different levels (personal, interpersonal and organizational).

In particular, our findings show that the risks of malaise in HCPs caring for COVID-19 patients are triggered by an extrinsic motivation, a static representation of the role (personal level), a bad relationship with collogues, patients, and family (interpersonal level), the perception of a lack of support from management, and an unclear/confused work purpose (organizational level). Additionally, the study shows that even in a situation of high stress there are resource factors that must be enhanced. We found that an intrinsic/ethical motivation, a flexible representation of one’s professional role (personal level), a good interpersonal relationship, the perception of a supportive leadership, and a sustainable and shared work purpose (organizational level) represented important protective factors. These protective and “mitigation” factors might play a crucial role in the well-being, care and effectiveness of professionals who work in healthcare system.

At the end of our work, we can highlight among the key protective factors described, the ability or not of management to support the working process through the predisposition of new practices and provide an emotional accompaniment to HCPs in particular during times of crisis.

The way forward for future studies concerns the role of human resources and in general the management needs in the healthcare contexts. From our study it emerges that participatory and cooperative processes seem to be increasingly needed to take care of our professionals. As a HCP suggested: *“We don’t know what the road is but if we try together we have a better chance of making it*”; this means that wherever no one has sure answers it is important to have the perception of being able to look for it together with others.

## Figures and Tables

**Table 1:** Risks Factors.

Risk Factors		
Personal level	Motivation	- Extrinsic motivation: concerns HCPs who were not entirely sure about pursuing a career as a doctor or nurse. It represented the trigger which brought to light some uncomfortable factors.
	Professional Role	- Static/traditional: HCPs who considered their job in a static and traditional way made a greater effort in dealing with the emergency.
Interpersonal level	Colleagues	- Dysfunctional relationship: HCPs with feeling of self-isolation and lack of support from colleagues were the most exposed to distress.
	Patients	- Emotional and relational distance: HCPs who were “too” close to the pain of patients and used inadequately the defense mechanisms were more exposed at risk of being overwhelmed.
	Family	- Bad relations: misunderstandings and tensions, generated especially by the situation of uncertainty (e.g., fear of contamination), occurred negatively affecting the well-being of HCPs.
Organizational level	Management	- Absence of support: the failure of the management to foster a good team climate and enhance professionals during the crisis led to a large number of negative emotions.
	Work purpose	- Undefined representation of the work purpose.

**Table 2:** Protective Factors.

Protective Factors		
Personal level	Motivation	- Intrinsic motivation: choice based on an inner desire and an individual propensity to care for others; - Ethic motivation: social and moral responsibilities and professional obligations.
	Professional role	- Dynamic/flexible: adaptability of the professionals to the different needs and functions required by the pandemic management.
Interpersonal level	Colleagues	- Solid relationship/collaboration: good relationships with colleagues constitute important and necessary sources of connection.
	Patients	- The feeling of gratification and the relationship with the patients constituted the driving force to go on. Some defense mechanisms helped HCPs to manage the huge emotional impact and the sufferings of the patients.
	Family	- Family and, more generally, loved ones represented a fundamental source of support for the HCPs.
Organizational level	Management	- Supportive and protected management. The management was able to support and enhance professionals in carrying on the new tasks.
	Work purpose	- Sustainable and shared representation of the work purpose.

## Data Availability

The data presented in this study are available on request from the corresponding author. The data are not publicly available due to privacy issue.
